# American Medical Association

**Published:** 1889-07

**Authors:** 


### American Medical Association.

The fortieth annual session of this body was held in Newport, R. I., beginning June 25th and continuing four days. It was an unusually interesting session. And now we can not do better than to quote from one who was present, viz., the Editor of the Cincinnati *Lancet and Clinic.* In this he says: " the attendance was hardly up to the anticipation of most of the members, which may be accounted for through various reasons. The representation from New York was very small on account of the lukewarmness of the profession as brought about through the new code business. A good delegation from Philadelphia and other parts of Pennsylvania was present, while New England was scarcely fairly represented in point of numbers. Comparatively few were present from the great West, St. Louis having but a single representative; all Southern Indiana had a single delegate, and St. Paul one, with but four or five from Chicago. Cincinnati and Louisville were fairly well represented. This sparse attendance from the West was wholly due to the discrimination of the railroads against the association, by the requirement of full-rate fares for going and returning.

The association was warmly welcomed by Dr. Storer and Governor Ladd, and the provision for amusement and entertainment of members was all that could be desired. Newport is full of historic associations, and is made up of most beautiful villas. The drives are superb. It is an ideal little city by the sea.

The profession was represented here by many of its most distinguished members. Even the venerable Dr. Davis, of Chicago, was present, as also Governor Gorcelon, of Maine, who, by the way, informed me that he graduated at the Ohio Medical College fifty years ago. Which reminds us that in medicine as in politics the Ohio men are not only numerous, but right at the front.

Surgeon Neilson, of the U. S. N., is stationed here; he is an Ohio man, and welcomed us royally.

Dr. Briggs, of Nashville, was there ; Owen, of Evansville ; Larrabee, Matthews, and Roberts, of Louisville; McMiller, of Columbus ; Chapman, of Toledo ; Comegys, Connor, Thrasher, W. H. Taylor, Dandridge, Christopher, Cleveland, Ayers, Fackler, French, Culbertson, and the noblest Roman of all, our beloved Dawson."

The work done in the various sections is said to have been the most thorough and efficient that has ever been done in the history of the association. The papers presented were all through exceptionally good.

Of the dental section, one who was present says: "It was relatively as well attended as any other section. Our papers were very good indeed; not a poor paper except mine. The exhibits of Drs. Sudduth and Andrews, illustrating upon the screen various histological subjects were of a high order and made a most excellent impression."

The next meeting is to be held at Nashville, Tenn., on the third Tuesday of May, 1890. Dr. E. M. Moore, of New York, was elected President of the association. Dr. J. Williams, of Boston was elected President of the Section of Dental and Oral surgery, and Dr. E. S. Talbott was re-elected Secretary of the section. We will give at least some of the papers read at the dental section in subsequent numbers of the REGISTER.

